# Targeted therapy for idiopathic pulmonary fibrosis: a bibliometric analysis of 2004–2024

**DOI:** 10.3389/fmed.2025.1543571

**Published:** 2025-03-20

**Authors:** Xinlei Zhang, Zengze Yuan, Xiawei Shi, Junchao Yang

**Affiliations:** ^1^The First Affiliated Hospital of Zhejiang Chinese Medical University, Zhejiang Provincial Hospital of Chinese Medicine, Hangzhou, Zhejiang, China; ^2^The Second Affiliated Hospital of Zhejiang Chinese Medical University, Hangzhou, Zhejiang, China

**Keywords:** idiopathic pulmonary fibrosis, targeted therapy, bibliometric analysis, transforming growth factor β, autotaxin inhibitor

## Abstract

**Background:**

Idiopathic pulmonary fibrosis (IPF) is a progressive and irreversible interstitial lung disease characterized by high mortality rates. An expanding body of evidence highlights the critical role of targeted therapies in the management of IPF. Nevertheless, there is a paucity of bibliometric studies that have comprehensively assessed this domain. This study seeks to examine global literature production and research trends related to targeted therapies for IPF.

**Method:**

A literature search was conducted using the Web of Science Core Collection, encompassing publications from 2004 to 2024, focusing on targeted therapies for IPF. The bibliometric analysis utilized tools such as VOSviewer, CiteSpace, and the “bibliometrix” package in R.

**Results:**

A total of 2,779 papers were included in the analysis, demonstrating a general trend of continuous growth in the number of publications over time. The United States contributed the highest number of publications, totaling 1,052, while France achieved the highest average citation rate at 75.74. The University of Michigan Medical School was the leading institution in terms of publication output, with 88 papers. Principal Investigator Naftali Kaminski was identified as the most prolific researcher in the field. The American Journal of Respiratory Cell and Molecular Biology emerged as the journal with the highest number of publications, featuring 98 articles. In recent years, the research has emerged surrounding targeted therapies for IPF, particularly focusing on agents such as TGF-β, pathogenesis, and autotaxin inhibitor.

**Conclusion:**

In this bibliometric study, we systematically analyze research trends related to targeted therapies for IPF, elucidating recent research frontiers and emerging directions. The selected keywords-idiopathic pulmonary fibrosis, targeted therapy, bibliometric analysis, transforming growth factor β, and autotaxin inhibitor—capture the essential aspects of this research domain. This analysis serves as a reference point for future investigations into targeted therapies.

## Introduction

Idiopathic pulmonary fibrosis (IPF) is a chronic, progressive, and incurable interstitial lung disease of unknown etiology, predominantly affecting middle-aged and elderly individuals, and is associated with a poor prognosis. The primary clinical manifestations include progressive dyspnea and reduced lung compliance (1). IPF is characterized by the destruction of normal lung architecture, persistent production and activation of myofibroblasts, and excessive accumulation of extracellular matrix, ultimately resulting in pulmonary fibrosis (2). The disease is estimated to affect ~3 million individuals globally, with its incidence on the rise and a high mortality rate and a median survival of 2–5 years after diagnosis (3–5). Currently, lung transplantation is regarded as the only effective treatment for IPF, but the therapy is only applicable to a small number of patients due to limitations in donor organ availability and chronic allogeneic rejection (6). The discovery of therapeutic targets highly relevant to pulmonary fibrosis and the development of effective antifibrotic therapies against these targets have been ongoing research priorities.

At present, pirfenidone and nintedanib are the only antifibrotic medications authorized for use in clinical settings (7). Pirfenidone works by decreasing the expression and activity of transforming growth factor β (TGF-β), which in turn lessens fibroblast activation and collagen production while also mitigating lung inflammation through inhibition of pro-inflammatory cytokines and chemokines. Nintedanib, a tyrosine kinase inhibitor, targets receptors associated with fibrosis (8). Although both medications help slow the decline in lung function and extend survival in individuals with IPF (9–11), they do not reverse the disease and have side effects such as gastrointestinal reactions (12). Therefore, the development of new anti-pulmonary fibrosis drugs is imperative. Over the past few years, many new therapeutic targets and drugs have emerged in clinical trials for IPF therapy. For example, dysregulation of phosphodiesterase 4B (PDE4B) can cause inflammation and fibrosis through hyperproliferation and activation. BI1015550, a novel PDE4B-selective inhibitor, has entered a phase III clinical study and prevented the decline of lung function in patients with IPF within 12 weeks (13). In all, current medications for IPF do not reverse the disease; novel targets and targeted therapeutic agents for IPF are being explored to improve efficacy, and adherence to the principles of precision medicine is urgent.

Bibliometrics is a discipline that studies the characteristics of literature and utilizes statistical methods to analyze it statistically (14). Bibliometric analysis facilitates a systematic approach to interpreting extensive volumes of unstructured data, enabling the elucidation and representation of the accumulated scientific knowledge and developmental intricacies within a well-established field. Currently, although a large number of studies have thoroughly researched and reviewed targeted therapies for IPF, there is currently no relevant literature that systematically analyzes and compares the field. This study aimed to offer a comprehensive bibliometric examination of publications and pertinent data concerning targeted therapies for IPF during the past 20 years (from January 1, 2004, to September 27, 2024) in order to evaluate the present, the current foci or hotspots and predict next research trends in the field.

## Methods

### Data searching and literature screening

In this research, the Science Citation Index Expanded (SCIE 1999–present) of Clarivate Analytics's Web of Science Core Collection (WoSCC) was utilized as the primary data source for conducting a systematic search and extracting data from literature published between 2004 and 2024. The searching strategy was formulated with reference to previous researches and the searching strategy was shown as follows: topic = (“Targeted Therapy” OR “Targeted” OR “Molecular Targeted Therapies” OR “Molecular Targeted” OR “Therapeutic Targets” OR “Targeted Drugs”) AND topic = (“IPF” OR “Idiopathic pulmonary fibrosis”). The search was carried out on September 27, 2024, covering the period from January 1, 2004, to that same date. To eliminate potential bias from daily database updates, all searches and downloads were completed within a single day. This process resulted in the retrieval of 2,913 relevant articles. After excluding 5 non-English articles and restricting the article types to theses and reviews to ensure the quality of the studies, a total of 2,779 relevant articles (2,014 theses and 765 reviews) were included, excluding meeting abstracts, editorial material, proceeding papers, early access, etc.

### Data extraction and analysis

We conducted an analysis of the WoSCC database to evaluate bibliometric indicators such as annual publications, countries, institutions, authors, journals, citations, and keywords. Utilizing Microsoft Excel 2021, we performed quantitative analyses to determine the yearly publication totals and the average citations per paper. Additionally, we assessed both the annual and cumulative publication counts for each country, as well as the overall number of papers authored by institutions, authors, and journals, in order to evaluate the quality of the publications. To evaluate the quality of scientific information, we primarily relied on the impact factor (IF) and category data provided by the Journal Citation Reports (JCR) for the year 2023. In some cases, we also used the H index to assess the scholarly achievements of countries, institutions, journals, and researchers. The H index refers to the fact that a research researcher has published at least H papers and each paper has been cited at least H times. The H index strikes a balance between the quantity and quality of the papers (represented by the number of citations), and it is an effective measure of the scholarly impact and scientific output of researchers, countries, institutions, and journals (15). For the visualization analysis, we employed VOSviewer [version 5.8 R3; (16)], CiteSpace [version 5.8 R3; (17)], and the R package “bibliometrix” [version 3.2.1; https://www.bibliometrix.org; (18)] to extract and examine relevant information from the gathered data and to create visual representations. Specifically, VOSviewer was utilized to perform keyword co-occurrence analysis as well as co-authorship and co-citation analyses pertaining to nations, organizations, authors, and journals.

CiteSpace V (version 5.8 R3) was utilized to produce bi-graphic overlays for the journals. Additionally, the R package “bibliometrix” (version 3.2.1; https://www.bibliometrix.org) facilitated the analysis of keyword evolution and the development of global distribution networks. In the resulting visualizations, various colors indicate clusters assigned to journals through clustering methods, with each cluster representing distinct research domains. Nodes symbolize countries, institutions, authors, and similar entities, while the connections between these nodes illustrate collaborative relationships. The dimensions of each node reflect the quantity of entities it represents, whereas the thickness of the lines connecting the nodes indicates the strength of collaboration among them. Total Link Strength (TLS) quantifies the cumulative strength of co-authorships and co-citations among countries, institutions, and authors.

## Results

### Trends of publication outputs and citations

**Figure 1:**
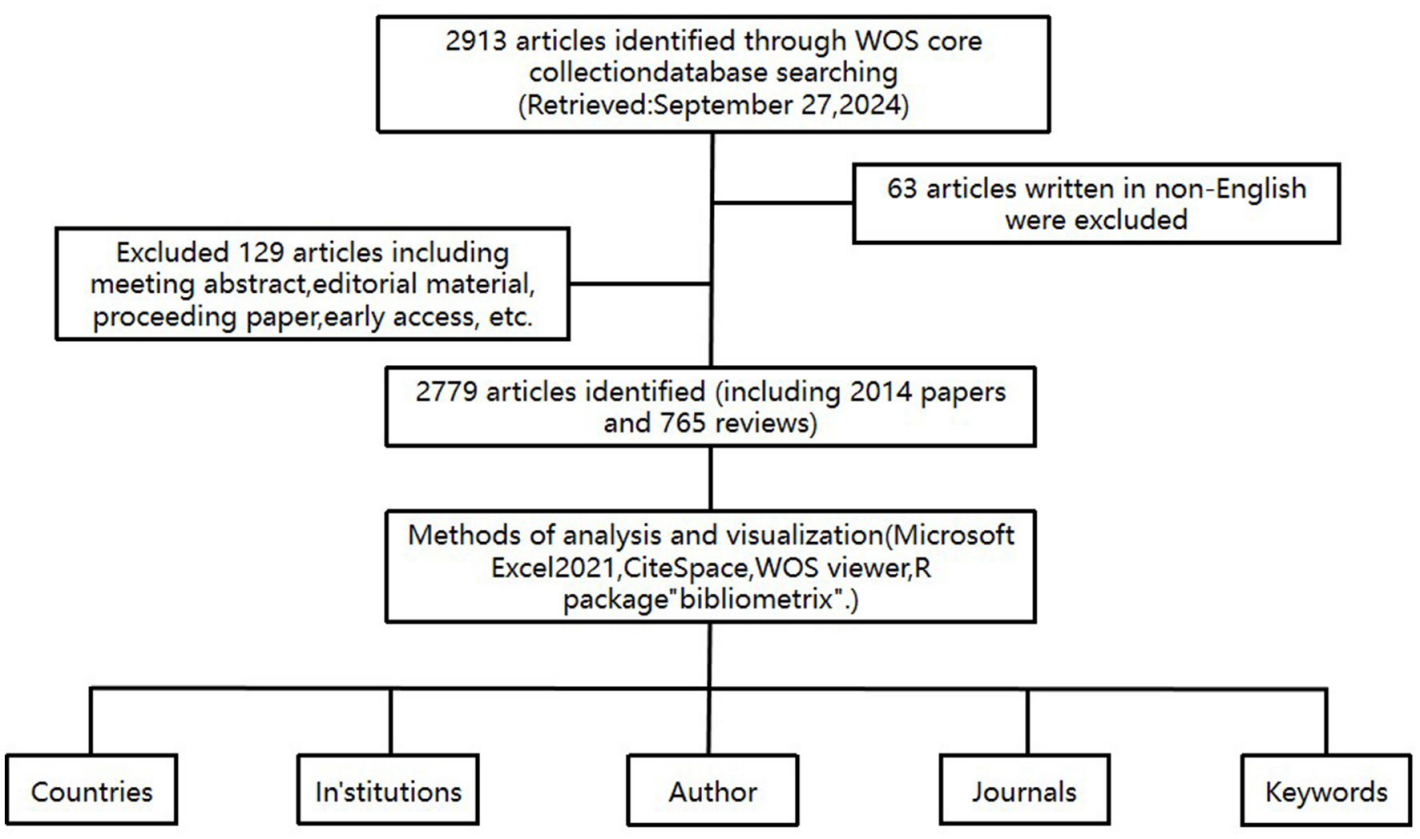
Flowchart of the publication's selection in the study.

**Figure 2:**
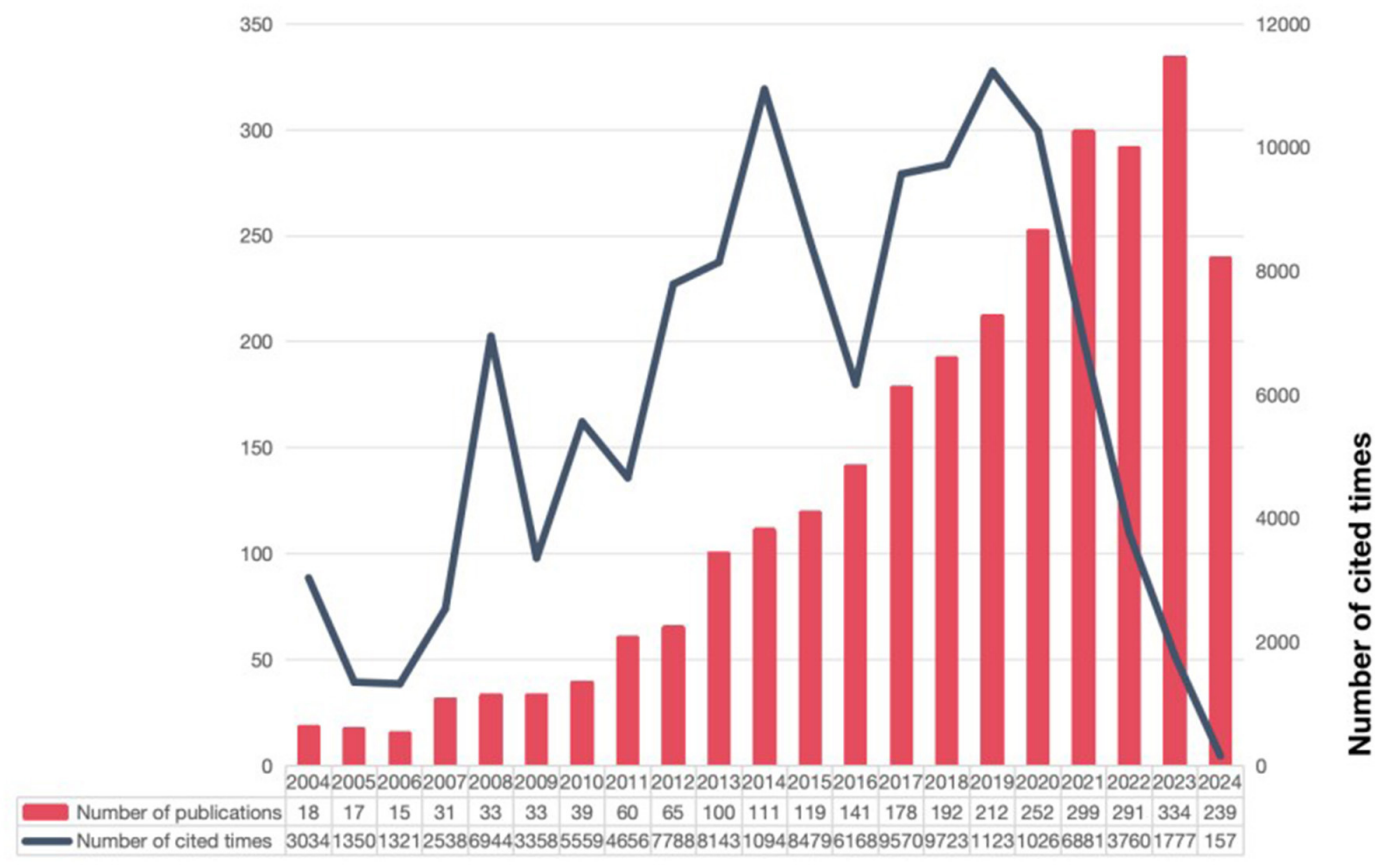
The number of global research publications on targeted therapy for IPF has shown a steadily increasing trend, with cumulative citations of all publications reaching 123,644 as of the search date.

According to the search strategy and screening process, we collected 2,779 literatures related to targeted therapy for IPF from WOSCC database, including 2,014 papers and 765 reviews (Figure 1). As shown in Figure 2, the number of research articles on targeted therapy for IPF showed a steadily increasing trend. As of the search date, the cumulative citations of all publications amounted to 123,644, yielding an average of 44.49 citations per document. We noticed that the number of annual citations displayed a tendency of fluctuating, increasing from 2004 to 2019, whereas it showed a decline from 2019 to 2024, with a peak of 11,230 citations in 2019. Additionally, the H index also declined in a progressive manner, even though the number of annual publications continued to grow.

### Contributions of countries/regions analysis

**Figure 3:**
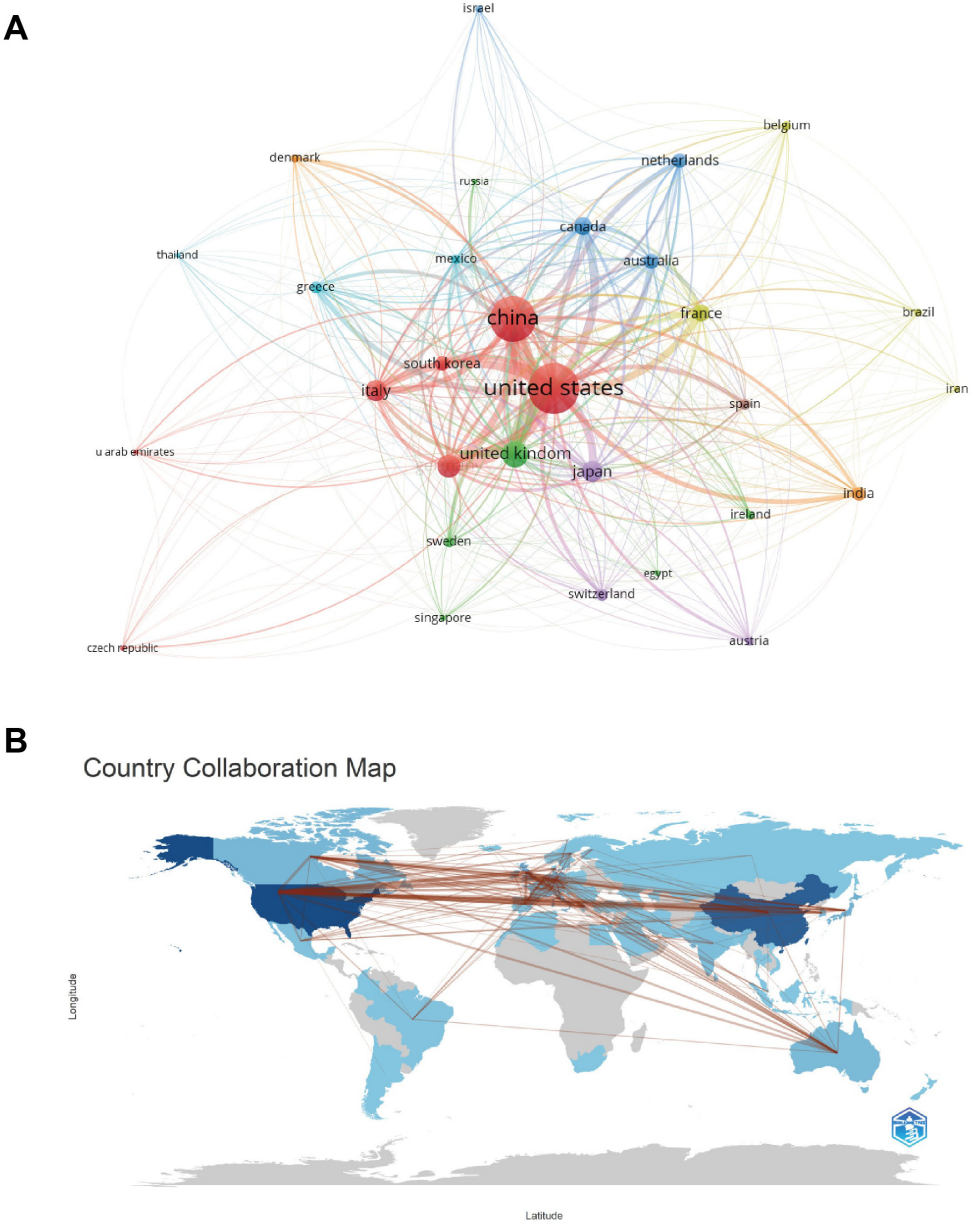
**(A)** A national citation visualization network map among countries, generated using VOSviewer version 5.8 R3, illustrates the citation relationships in the literature. **(B)** A cross-country/region collaboration visualization map highlights the active cooperative relationships between different countries in this research field.

Table 1 lists the top 10 productive countries on targeted therapy for IPF from 2004 to 2024. The United States possesses the most quantity of documents, including 37.86% of the total (1,052/2,779), succeeded by China (30.30%, 842/2,779) and the United Kingdom (9.43%, 262/2,779). Total citations and h-index of the United States were several times ahead of any other country, with 75,530 and 129, respectively, indicating that the United States was the most cutting-edge country in this field. Indeed, the average citation frequency reflects the quality of a paper to a large extent, and the highest average citation is in France (75.74), followed by Canada (75.61) and Germany (72.81). The average number of citations for papers in the United States, China, and the United Kingdom are 71.80, 21.48, and 68.71, respectively. China ranks second in total citations among the 10 countries, but last in the average number of citations for papers, which indicates that the quality of papers in China needs to be improved. Figure 3A illustrates the citation relationships among countries in the literature. The United States is the leading country in TLS, with a value of 13,063, followed by China at 7,818 and the United Kingdom at 4,238. Subsequently, we utilized the R package “bibliometrix” to construct a geographic distribution map based on the number of papers and collaborations in each country (Figure 3B), which represents the active cooperative relationship between different countries in this research area.

**Table 1:** The top 10 productive countries on targeted therapy for IPF.

**Rank**	**Countries**	**Article count**	**Percentage (n/2,779)**	**H-index**	**TLS**	**Total citations**	**Average citation per article**
1	United States	1,052	0.3786	129	13,063	75,530	71.80
2	China	842	0.3030	65	7,818	18,082	21.48
3	United Kingdom	262	0.0943	63	4,238	18,002	68.71
4	Germany	202	0.0727	58	4,210	14,707	72.81
5	Japan	180	0.0648	44	2,383	8,875	49.31
6	Italy	167	0.0601	42	2,729	7,008	41.96
7	Canada	117	0.0421	38	2,542	8,846	75.61
8	France	114	0.0410	41	2,135	8,634	75.74
9	Australia	92	0.0331	37	1,637	4,477	48.66
10	South Korea	82	0.0295	23	1,382	5,071	61.84

### Analysis of institutions

Table 2 presents the 10 leading institutions ranked by their publication volume, and the majority of these organizations are based in the United States. The University of Michigan Medical School tops the list with the highest number of published articles, followed closely by the University of Pittsburgh and the University of Alabama at Birmingham. Additionally, the University of Michigan Medical School boasts the highest H index at 43, while Imperial College London has achieved the highest average citation count per article, with 123.60 citations.

**Table 2:** The top 10 productive institutions ranked by the numbers of publications.

**Rank**	**Institutions**	**Countries**	**Article count**	**H-index**	**Total citations**	**Average citation per article**
1	University of Michigan Medical School	United States	88	43	7,392	84.00
2	University of Pittsburgh	United States	73	36	5,764	78.96
3	The University of Alabama at Birmingham	United States	58	32	4,810	82.93
4	Imperial College London	United Kingdom	55	32	6,798	123.60
5	Xiangya Hospital Central South University	China	46	16	1,127	24.50
6	University of Colorado Anschutz Medical Campus	United States	43	27	3,194	74.28
7	Medical School of Nanjing University	China	32	14	886	27.69
8	Vanderbilt University Medical Center	United States	31	21	2,754	88.84
9	Yale University School of Medicine	United States	30	18	1,272	42.40
10	Faculty of Health Sciences, MacMaster University	Canada	29	15	902	31.10

**Figure 4:**
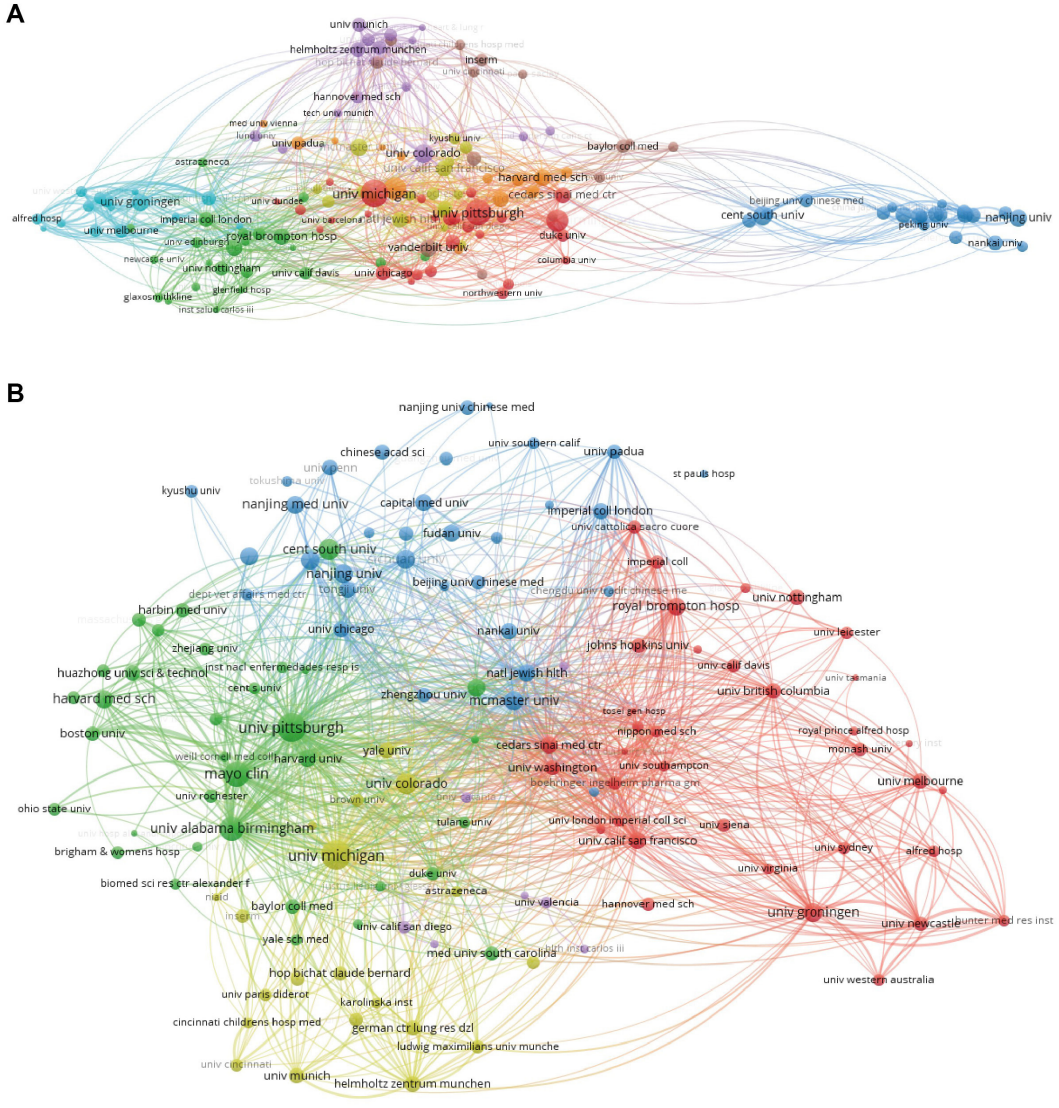
**(A)** A cross-institutional collaboration visualization network map, generated using VOSviewer version 5.8 R3, illustrates the intricate interconnections within each cluster. **(B)** An institutional citation analysis network diagram reveals the complex partnerships and citation relationships among global institutions.

Close inter-agency collaboration has led to the involvement of more organizations in this area. Figure 4A summarized 150 entries and 1,636 links, with the 147 entries color-coded into eight clusters. Institutions within each cluster are intricately interconnected. Figure 4B presents a citation analysis network diagram including 130 items and 6,828 linkages. The University of Michigan possesses the highest TLS at 1,119, underscoring its substantial connectedness inside the network. The above results suggested the complex partnerships and citations between global institutions.

### Analysis of the active authors and co-cited authors

**Figure 5:**
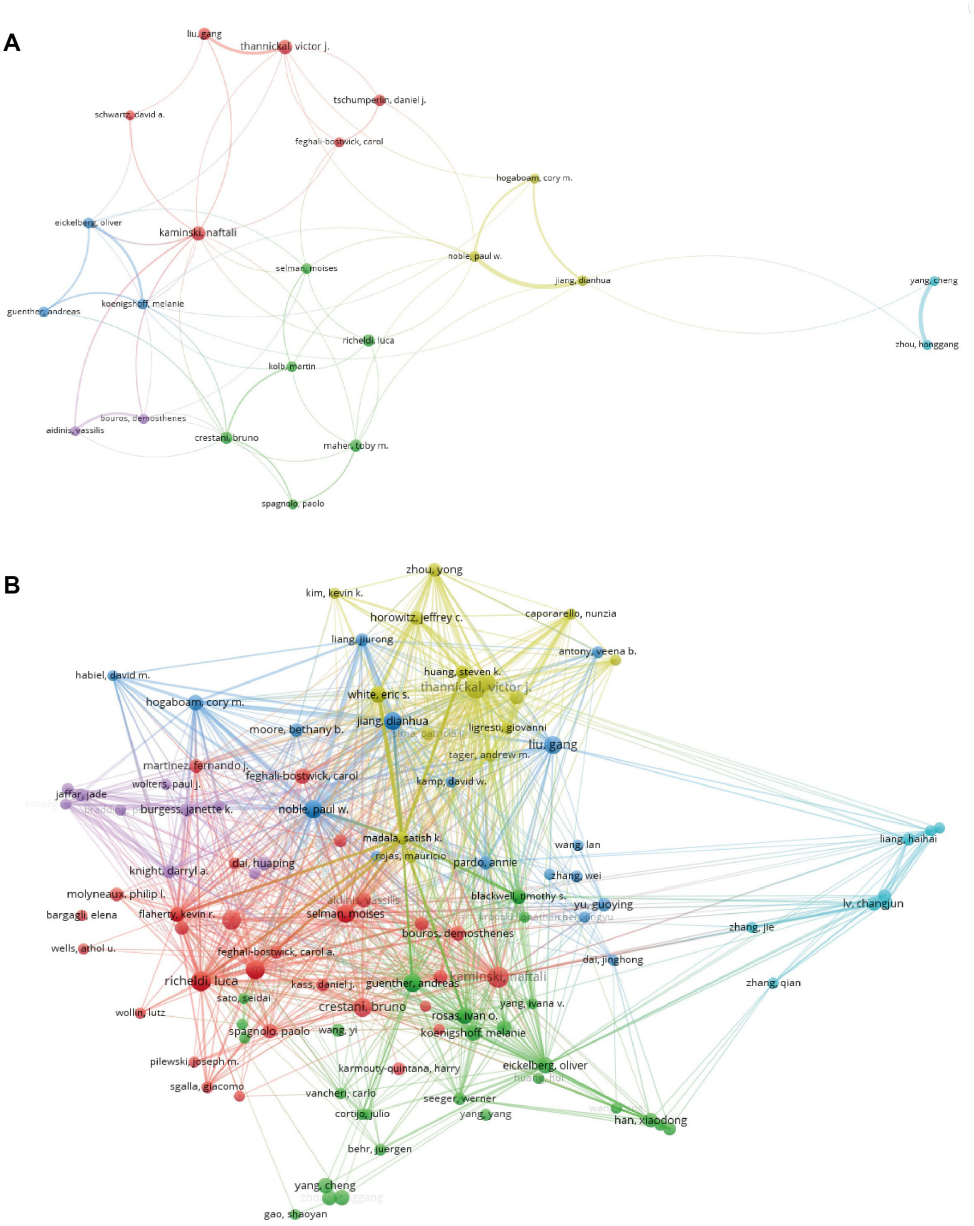
**(A)** The co-authorship analysis network visualization map, generated using VOSviewer version 5.8 R3. **(B)** A graphical representation of the authors' collaborative citation network, created using VOSviewer version 5.8 R3.

Table 3 lists the top 10 most productive authors, and these authors were mostly from the United States. Notably, Kaminski, Naftali is the author with the most publications, with 38 articles and an H index of 27. While Paul W. published fewer papers, his total citations were as high as 5,404 and is the author with the highest number of citations per article on average with 245.64 citations. Note that both individuals were from the United States. Figure 5A visualizes the map of author co-authorship analysis generated by VOSviewer. It is worth noting that Crestani, Bruno, a prolific figure in the field, has a much wider network of collaborators. Figure 5B illustrates a graphical representation of the authors' collaborative citation network, including 106 entries, 6 clusters, and 3,411 links. The authors with the highest TLSs include Noble, Paul W. (TLS = 998), Thannickal, victor j. (TLS = 808), and Selman, moises (TLS = 753).

**Table 3:** The top 10 most productive authors on targeted therapy for IPF.

**Rank**	**Author**	**Article count**	**H-index**	**Countries**	**Total citations**	**Average citation per article**	**Institutions**
1	Kaminski, Naftali	38	27	United States	3,229	84.97	Yale University
2	Crestani, Bruno	33	20	France	1,525	46.21	Hopital Universitaire Bichat-Claude Bernard
3	Feghali-Bostwick, Carol	31	24	United States	2,992	96.52	University of South Carolina School of Medicine
4	Kolb, Martin	29	17	Canada	3,872	133.52	McMaster University
5	Richeldi, Luca	28	13	Italy	3,881	138.61	Catholic University of the Sacred Heart
6	Maher, Toby M.	27	21	United States	1,906	70.59	University of Southern California
7	Koenigshoff, Melanie	25	20	United States	2,396	95.84	University of Pittsburgh
8	Tschumperlin, Daniel J.	23	16	United States	2,365	102.83	Mayo Clinic
9	Noble, Paul W.	22	17	United States	5,404	245.64	Cedars Sinai Medical Center
10	Hogaboam, Cory M.	22	16	United States	1,383	62.86	Cedars Sinai Medical Center

### Analysis of top journals and co-cited journals

Table 4 lists the top 10 journals that have published the highest number of articles in this research area. As shown in the table, as of September 2024, the top 10 journals have published a total of 567 articles, representing 20.40% of the included literature. American Journal of Respiratory Cell and Molecular Biology (IF 2023 = 5.9) had the highest number of articles with 98, followed by International Journal of Molecular Sciences (IF 2023 = 4.9) and American Journal of Molecular Sciences (IF 2023 = 4.9) and American Journal of Physiology-Lung Cellular and Molecular Physiology (IF 2023 = 3.6). Among the top 10 journals, 4 originate from the United States, 3 from the United Kingdom, and 3 from Switzerland. The American Journal of Respiratory and Critical Care Medicine possesses the greatest H-index (43), total citations (7,184), and impact factor (IF 2023 = 19.3).

**Table 4:** The top 10 journals related to the research of IPF targeted therapy ranked by publication number.

**Rank**	**Journal Title**	**Country**	**Count**	**IF (2023)**	**Quartile in category (2023)**	**H-index**	**Total citations**
1	American Journal of Respiratory Cell and Molecular Biology	United States	98	5.9	Q1/2	42	5,120
2	International Journal of Molecular Sciences	Switzerland	65	4.9	Q1/2	23	1,707
3	American Journal of Physiology-Lung Cellular and Molecular Physiology	United States	62	3.6	Q1/2	29	3,375
4	American Journal of Respiratory and Critical Care Medicine	United States	60	19.3	Q1/1	43	7,184
5	Respiratory Research	United Kingdom	59	4.7	Q1/2	21	1,926
6	PloS One	United States	57	2.9	Q1/3	28	2,827
7	Science Progress	United Kingdom	48	2.6	Q2/4	21	1,153
8	Frontiers in Pharmacology	Switzerland	46	4.4	Q1/2	18	1,653
9	Frontiers in Immunology	Switzerland	40	5.7	Q1/2	15	710
10	European Respiratory Journal	United Kingdom	32	16.6	Q1/1	24	2,697

**Figure 6:**
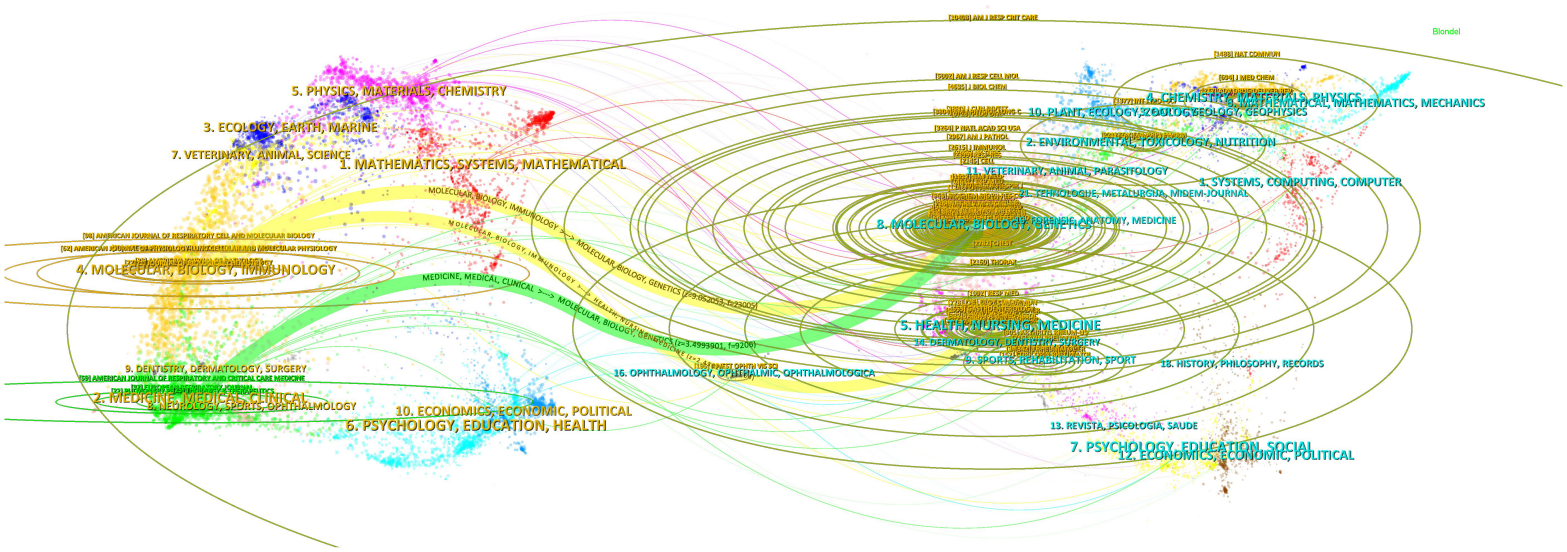
A dual-map overlap of journals on IPF targeted therapy (drawn by CiteSpace V version5.8 R3). The left side of the figure illustrates the domain of the administering literature (applied research), while the right side depicts the domain of the cited literature (basic research). Various colors denote distinct citation trajectories, which subsequently illustrate the causal linkages among the citations.

Figure 6 visualizes the linkage of related journals by overlaying a double figure. The left side of the figure illustrates the domain of the administering literature (applied research), while the right side depicts the domain of the cited literature (basic research). Various colors denote distinct citation trajectories, which subsequently illustrate the causal linkages among the citations. Three main citation paths are identified in this viewable view, including one green path and two yellow paths. The yellow routes signify that the provided citations predominantly pertain to the domains of molecules, biology, and immunology, whereas the cited references primarily relate to the subjects of molecules, biology, genetics, health, nursing, and medicine. The green path signifies that the sizing literature is predominantly disseminated throughout the domains of medicine, medical, and clinical fields, whereas the cited literature is primarily concentrated in the areas of molecular, biology, and genetics. Furthermore, the elliptical curves on the periphery illustrate the extent of effect of the referenced material in the domain.

### Analysis of citations and co-cited citations

**Figure 7:**
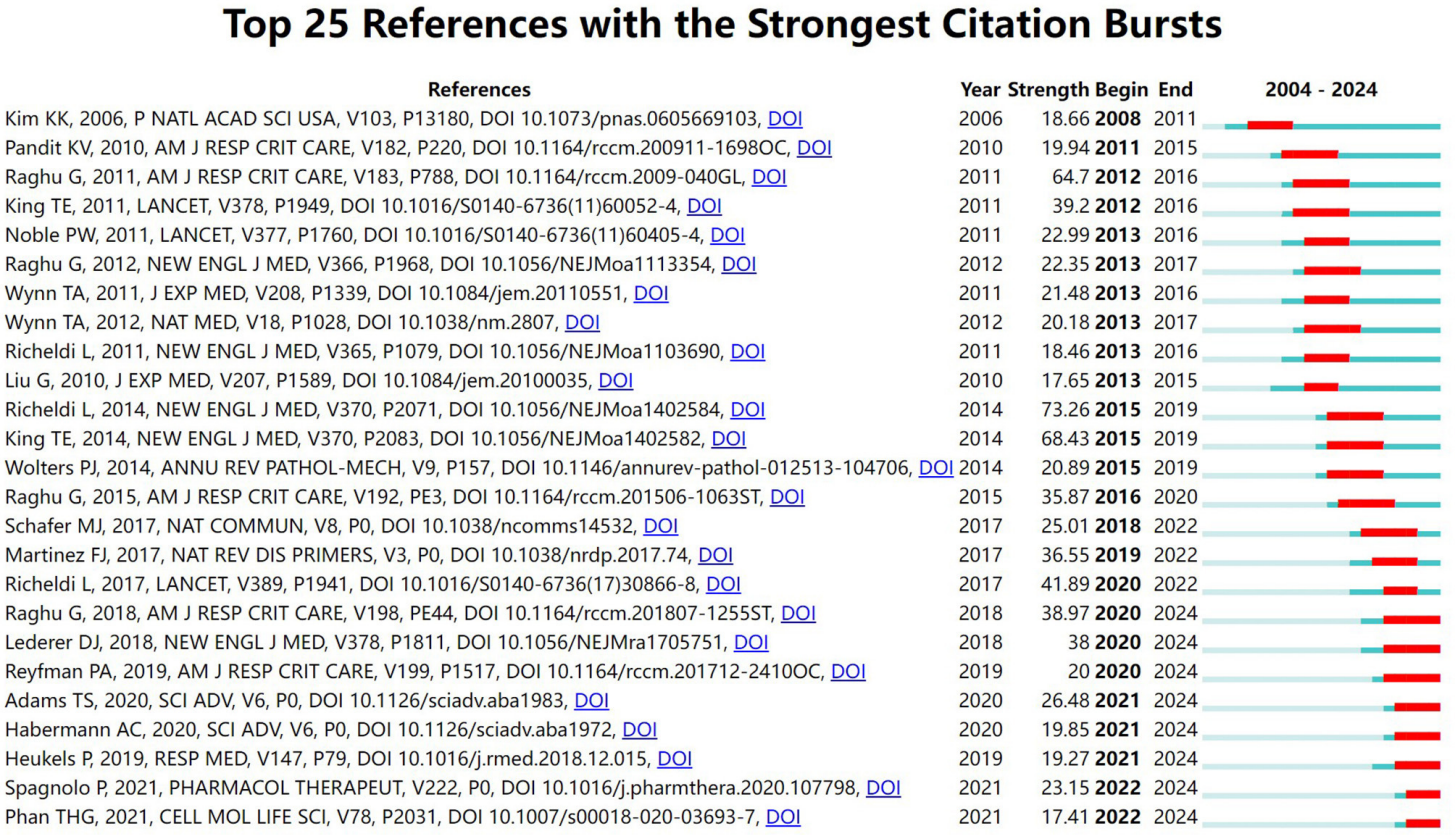
Top 25 references with the strongest citation bursts on IPF targeted therapy (The green line segment represents the time interval, and the red line segment represents the active time).

Table 5 lists the top 10 most cited articles in the field. It is worth noting that Nature is an important source of significant contributions to the field, with 40% of the top 10 most cited articles coming from Nature and its subpublications, respectively. All of the references in the top 10 were cited a total of 670 times or more, underscoring their far-reaching influence. The article by Wynn (43), published in the Journal of Pathology, was the most cited, with 3,214 citations. Figure 7 illustrates our investigation of citation burstiness utilizing CiteSpace V (version 5.8 R3), revealing a total of 25 papers exhibiting the highest citation burstiness. The literature featuring citation bursts initially emerged in 2008, with its inception traced back to a publication published in 2006. Seventy-six percent of these references underwent a citation surge from 2010 to 2020. The latest instance of a citation burst occurred in 2022 and continues to persist.

**Table 5:** The top 10 related articles with the most citations concerning the IPF targeted therapy.

**Title**	**First author**	**Journal**	**Year**	**Citations**	**Main conclusion**
Cellular and molecular mechanisms of fibrosis	Wynn, TA	Journal of Pathology	2008	3,214	This article reviews the cellular and molecular mechanisms of fibrosis, exploring in detail the role of fibroblasts, immune mechanisms, inflammatory factors, and signaling pathways in the fibrotic process. Novel mediators and pathways such as cytokines, chemokines and TLR antagonists, angiogenesis inhibitors, and TGF β signaling modulators could be new targets for the treatment of fibrosis (43).
Efficacy and Safety of Nintedanib in Idiopathic Pulmonary Fibrosis	Richeldi, L	New England Journal of Medicine	2014	3,111	Using two randomized, double-blind, placebo-controlled Phase III clinical trials (INPULSIS-1 and INPULSIS-2), the article found that nintedanib was effective in slowing the decline in FVC in patients with idiopathic pulmonary fibrosis, consistent with a slowing of disease progression. Common side effects included diarrhea, nausea, and vomiting. The overall safety profile was consistent with known information. The results of this study support nidanib as one of the treatment options for idiopathic pulmonary fibrosis (44).
Mechanisms of fibrosis: therapeutic translation for fibrotic disease	Wynn, TA	Nature Medicine	2012	2,458	This literature review examines the mechanisms of fibrosis and its therapeutic translation. Treatment of fibrosis requires a multi-pathway approach to control inflammation and cellular damage as well as to regulate ECM synthesis and degradation. Future studies should further explore the molecular mechanisms of fibrosis and develop targeted therapies to improve the prognosis of patients with fibrosis-related diseases (45).
Fibrotic disease and the TH1/TH2 paradigm	Wynn, TA	Nature Reviews Immunology	2004	1,304	This literature focuses on the relationship between fibrotic disease and the TH1/TH2 balance. The TH2 cellular response promotes fibrosis while promoting tissue repair, and the TH1 cellular response inhibits fibrosis while inhibiting tissue repair. Therapies that both utilize the beneficial aspects of the TH2 response (tissue repair) and prevent or inhibit its deleterious features (fibrosis) are targets for future research (46).
Cellular senescence mediates fibrotic pulmonary disease	Schafer, MJ	Nature Communications	2017	962	This paper found that senescent cells play an important role in the progression of fibrotic lung disease through modeling, senescent cell detection, and drug intervention. Targeted removal of senescent cells may be an effective strategy for the treatment of fibrotic lung disease (47).
Fibrosis: from mechanisms to medicines	Henderson, NC	Nature	2020	850	The article discusses transformative experimental strategies that are being used to dissect the key cellular and molecular mechanisms that regulate fibrosis, as well as translational approaches that enable patients with fibrosis to access precision medicine-based therapies (48).
Single-Cell Transcriptomic Analysis of Human Lung Provides Insights into the Pathobiology of Pulmonary Fibrosis	Reyfman, PA	American Journal of Respiratory and Critical Care Medicine	2019	733	This study demonstrated heterogeneity within alveolar macrophages and epithelial cells from patients with pulmonary fibrosis by single-cell RNA sequencing of lung tissue, bronchoscopic frozen biopsy samples from patients with idiopathic pulmonary fibrosis, providing hope for the discovery of multicellular pathways important to the pathogenesis of the disease as well as for generating or ruling out hypotheses regarding the function of the different populations of cells that appear during the disease (49).
Fibroblasts in fibrosis: novel roles and mediators	Kendall, RT	Frontiers in Pharmacology	2014	718	This review summarizes the functions of various fibroblasts and illustrates the central role of fibroblasts in the pathology of fibrosis. The production of giant extracellular matrix associated with fibrosis, myofibroblast differentiation, the role of fibroblasts in matrix-cancer interactions, and potential clinical therapies targeting fibroblasts are reviewed (50).
Senolytics in idiopathic pulmonary fibrosis: Results from a first-in-human, open-label, pilot study	Justice, JN	Ebiomedicine	2019	693	This first-in-human, two-center, open-label study of an intermittent dasatinib plus quercetin intervention in IPF participants demonstrated that senolytics can alleviate physical dysfunction in IPF patients (51).
Monocyte-derived alveolar macrophages drive lung fibrosis and persist in the lung over the life span	Misharin, AV	Journal of Experimental Medicine	2017	672	This study developed a novel lineage tracking system in mice to unambiguously identify tissue-resident alveolar macrophages and monocyte-derived alveolar macrophages during fibrosis development and the subsequent life cycle of the animals, revealing significant heterogeneity in alveolar macrophage function during pulmonary fibrosis and demonstrating that selectively targeting alveolar macrophage differentiation within the lungs can ameliorate fibrosis without the adverse consequences associated with overall monocyte or adverse consequences associated with depletion of tissue-resident alveolar macrophages (52).

### Keywords analysis of research hotspots

**Figure 8:**
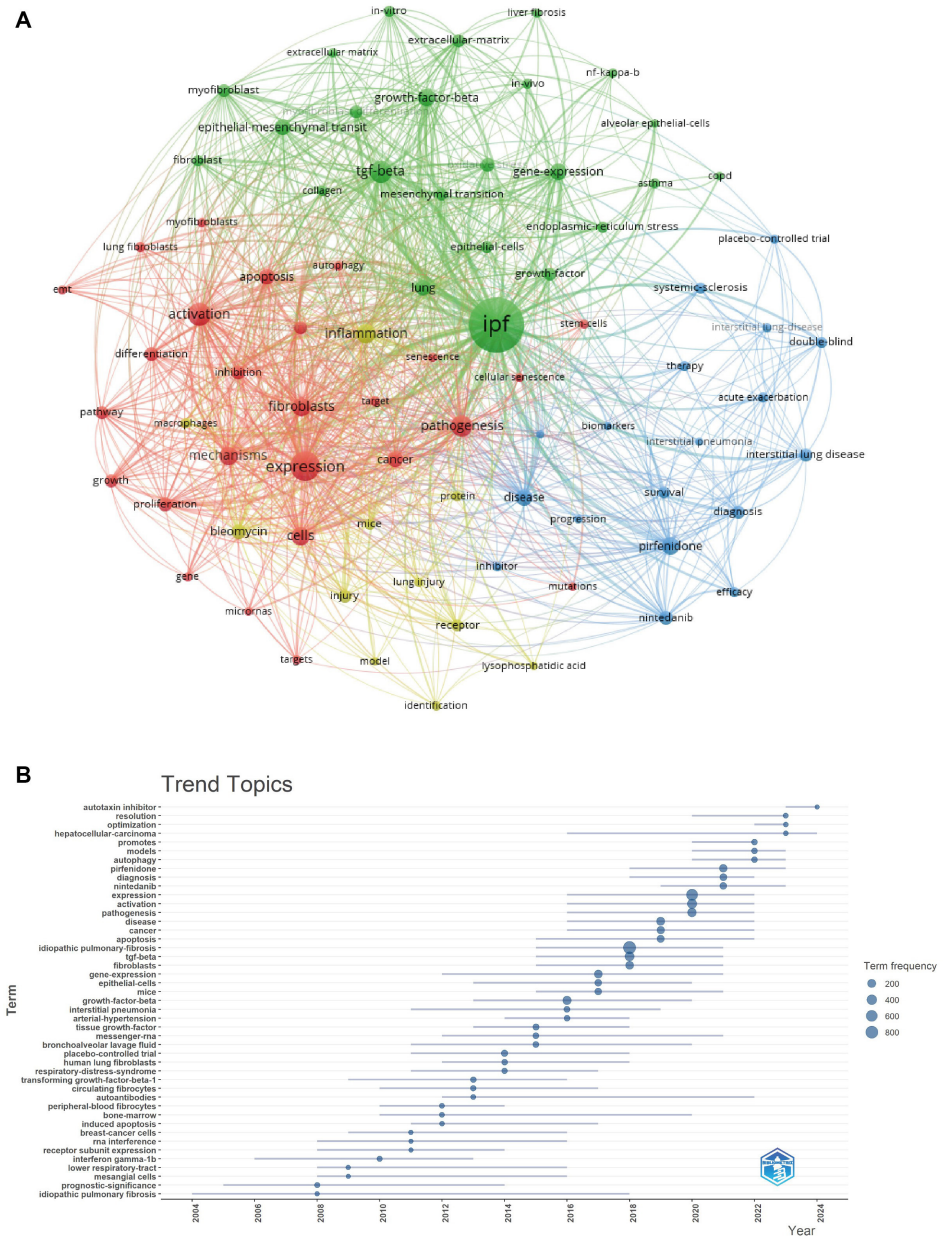
**(A)** The keyword co-occurrence network visualization map, generated using VOSviewer version 5.8 R3, categorizes keywords into distinct clusters, each represented by a unique color (clusters 1–4 are shown in green, red, yellow, and blue). The interconnections among these keywords form a network comprising 2,671 links. **(B)** A comprehensive thematic analysis of keyword trends was conducted using the R package “bibliometrix.” The results reveal a significant evolution in research themes over time, and suggest that these keywords likely represent emerging research focal points within the field of targeted therapies for IPF.

We meticulously extracted keywords from the titles and abstracts of 2,779 articles included in our analysis. Subsequently, we employed the VOS browser (version 5.8 R3) for both visualization and examination, concentrating on keywords that appeared more than 50 times. As illustrated in Figure 8A, a comprehensive cluster analysis followed, leading to the identification of 79 frequently occurring keywords. These keywords were categorized into distinct clusters, each illustrated by a unique color (clusters 1–4 are depicted in green, red, yellow, and blue). The relationships among these keywords created a network consisting of 2,671 connections.

To refine our analysis, we conducted a thorough thematic examination of keyword trends utilizing the R package “bibliometrix.” The findings of this analysis indicate a notable evolution in themes over the years (Figure 8B). From 2009, prevailing research keywords included growth factor β, anti-interferon gamma-1b, factor alpha, and peripheral blood fibrocytes. Following 2016, there was a substantial rise in topics concerning fibroblasts, nintedanib, and pirfenidone. Furthermore, the terms “optimization,” “resolution,” and “autotaxin inhibitors” have experienced a significant increase in usage over the past 1–4 years. This upward trend indicates that these keywords likely highlight current research focal points within the domain of targeted therapies for IPF.

## Discussion

In this study, bibliometric techniques were utilized to perform an extensive analysis of the global literature concerning targeted therapy for IPF spanning the period from 2004 to 2024. The results reveal a consistent upward trend in the volume of publications over the years, a pattern projected to persist into the future. From 2004 to 2019, the total number of citations exhibited a fluctuating yet generally upward trajectory. However, post-2019, there was a notable decline in both the aggregate citation count and the H-index of articles within this field. This downturn may suggest a potential decline in the impact or influence of research in this area. While it is essential to consider potential factors such as citation lag, there is an urgent necessity to identify novel research focal points in this domain to rejuvenate and enhance the quality of findings within related disciplines.

A comprehensive analysis of global academic publication trends indicates that the United States contributes most significantly to the field. The United States not only produces the highest volume of publications but also excels in metrics such as the H Index, Total Link Strength (TLS), and Total Citation Count. These indicators underscore the superior quality and extensive impact of the United States' scholarly output. China and the United Kingdom closely follow. Despite China securing the second position in terms of the number of publications, total link strength (TLS), and total citations, the average citation count per paper remains relatively low. This suggests that Chinese scholars should prioritize enhancing the quality of their academic publications to increase the international impact of their research.

Among the top 10 research institutions, the United States comprises 66% of this group based on the volume of published papers, underscoring its dominance in the relevant research field. China follows with 12%. However, it is important to recognize the achievements and status of other developing research institutions, as they play a significant role in enhancing the academic influence of their nations. Acknowledging the contributions of these emerging institutions is vital for improving the scholarly reputation of their respective countries.

The correlation analysis of highly productive scholars reveals that 7 of the top 10 scholars are from the United States, identified as having the highest H-index, total citations of their articles, and average citations per article. Notable authors include Kaminski, Naftali from Yale University, Crestani, Bruno from Hopital Universitaire Bichat-Claude Bernard, and Feghali-Bostwick, Carol from the University of South Carolina School of Medicine, who are recognized as major contributors to the field. A visual comparison and analysis of co-authored publications provide insights into existing collaborations and help identify significant or prospective collaborators in the area. Furthermore, the present research depicts co-citation networks among authors, indicating that those sharing the same color are engaged in similar research domains. The size of each node corresponds to the influence and prominence of the respective researcher within their discipline. Notably, Crestani, Bruno and Richeldi, Luca exhibit extensive collaboration networks, while Noble, Paul W. has the most robust co-citation connections. These researchers play a crucial role in the study of targeted therapy for IPF, and their teams are expected to make substantial contributions to high-impact publications in related areas.

In the area of targeted therapy for IPF, leading publications like the American Journal of Respiratory and Critical Care Medicine, European Respiratory Journal, American Journal of Respiratory Cell and Molecular Biology, and Frontiers in Immunology have established themselves as key academic resources. These results offer essential insights for researchers, encouraging them to target these esteemed journals for their manuscript submissions and inspiring the dissemination of their research outcomes within these influential platforms. Importantly, among the top 10 journals in this field, 2 possess IF exceeding 10.0: the American Journal of Respiratory and Critical Care Medicine (IF 2023: 19.3) and the European Respiratory Journal (IF 2023: 16.6). Furthermore, there are two additional journals within this top tier that have IF ratings between 5.0 and 10.0, specifically the American Journal of Respiratory Cell and Molecular Biology (IF 2023: 5.9) and Frontiers in Immunology (IF 2023: 5.7). Overall, the pursuit of publication in high-impact journals within the domain of targeted therapy for IPF continues to present significant challenges.

In the domain of targeted therapy for idiopathic pulmonary fibrosis (IPF), prominent academic journals, including the American Journal of Respiratory and Critical Care Medicine, the European Respiratory Journal, the American Journal of Respiratory Cell and Molecular Biology, and Frontiers in Immunology, have established themselves as crucial scholarly resources. These journals offer invaluable insights to researchers, encouraging the submission of manuscripts to these esteemed platforms and promoting the dissemination of research findings within influential academic communities. Notably, among the top 10 journals in this field, 2 have impact factors (IF) exceeding 10.0: the American Journal of Respiratory and Critical Care Medicine (IF 2023: 19.3) and the European Respiratory Journal (IF 2023: 16.6). Additionally, there are two other journals within this elite tier that possess IF ratings between 5.0 and 10.0, specifically the American Journal of Respiratory Cell and Molecular Biology (IF 2023: 5.9) and Frontiers in Immunology (IF 2023: 5.7). Overall, the pursuit of publication in high-impact journals within the domain of targeted therapy for IPF continues to present significant challenges.

“Highly cited references” denote studies that have been frequently cited within a specified period. This metric indicates the research interest and dynamics surrounding these papers in the field of targeted therapies for idiopathic pulmonary fibrosis (IPF), highlighting their substantial impact on the scientific community during this timeframe. The initial surge in citations occurred in 2008, following a seminal publication by Kim et al. (19). This study provided evidence that alveolar epithelial cells serve as progenitors for fibroblasts *in vivo*, underscoring the significant regulatory function of the transient extracellular matrix in the transdifferentiation of epithelial cells during fibrogenesis. This conclusion was reached through experiments involving genetically engineered mice that express β-galactosidase exclusively in lung epithelial cells, which were subsequently monitored within a well-established pulmonary fibrosis model. The increasing number of citations further underscores the burgeoning scientific interest in developing targeted therapies for IPF.

The synthesis of pertinent keywords is crucial for efficiently understanding targeted therapies for idiopathic pulmonary fibrosis (IPF). By employing cluster analysis and thematic trend analysis of these keywords, we identified that research on targeted therapies for IPF is focused on several key domains. In the context of IPF, persistent harmful stimuli, coupled with the compromised function of the alveolar epithelium, disrupt the cytokine equilibrium in lung tissue. This modified microenvironment is marked by increased concentrations of pro-fibrotic substances, including platelet-derived growth factor (PDGF), fibroblast growth factor (FGF), and TGF-β (20). Since 2009, scholars have focused on TGF-β, which is a crucial factor in the pathogenesis for IPF. Following injury to epithelial cells, TGF-β acts as a significant pro-fibrotic agent that promotes the progression of pulmonary fibrosis (21). Its multifaceted roles are vital in the proliferation and differentiation of both epithelial cells and fibroblasts, while also stimulating the formation of myofibroblasts. This leads to an increased production of extracellular matrix (ECM), and the uncontrolled accumulation of ECM contributes to pulmonary sclerosis and impaired gas exchange, ultimately resulting in a decline in lung function in patients with IPF (22). Furthermore, TGF-β is implicated in promoting epithelial-mesenchymal transition (EMT), enhancing apoptosis and migration of epithelial cells, and inducing the synthesis of connective tissue growth factor (CTGF) along with other relevant mediators (23, 24). Therefore, TGF-β is a key target of IPF, and many targeted drugs achieve antifibrotic effects by inhibiting TGF-β signaling.

Currently, only pirfenidone and nintedanib are approved antifibrotic drugs for clinical use. Pirfenidone inhibits TGF-β signaling, thereby enhancing the efficacy of transplanted progenitor cells (25). Although the precise mechanism of action of nintedanib remains unclear, its effects on TGF-β signaling and atypical autophagy have been demonstrated (26). Furthermore, nintedanib effectively blocks the activation of key receptors, including the PDGF receptor, fibroblast growth factor receptor, vascular endothelial growth factor receptor, and Src-family kinases, all of which are implicated in the pathogenesis of IPF (27). In addition to nintedanib and pirfenidone, which have been approved for clinical use, recent advances have emerged in the development of drugs targeting novel mechanisms that inhibit TGF-β signaling. Integrins are known to facilitate the activation of TGF-β, with avβ6 integrins being among the most extensively studied potential therapeutic targets in IPF. However, a phase II clinical trial of an anti-integrin drug, BG00001, revealed no significant change in forced vital capacity (FVC) between the treatment group and the placebo group, and a subset of patients even experienced acute exacerbations (28). Besides avβ6, several other integrins, such as avβ1 and avβ3, also regulate TGF-β activity and promote fibroblast proliferation and growth, indicating that they may serve as important targets for future research.

Pentraxin 2 inhibits the production of TGF-β, likely through the inhibition of monocyte-to-macrophage differentiation, which subsequently reduces the levels of TGF-β. Zinpentraxin alfa (rhPTX-2) is a recombinant form of human pentraxin-2. In a phase II trial (NCT02550873), rhPTX-2 demonstrated significant efficacy in slowing the decline of forced vital capacity (FVC) and the 6-min walk distance [6 MWD; (29)]. However, in the 52-week phase 3 randomized controlled trial (RCT) STARSCAPE, no significant difference was observed between rhPTX-2 and placebo in patients with IPF (30). Galectin-3 (Gal-3) is a lectin that binds to β-galactosides and is significantly elevated in various fibrotic disorders. This β-galactoside-binding lectin promotes the development of fibrosis by modulating the expression of TGF-β receptors. A phase I/IIa trial demonstrated that the expression of Gal-3 was reduced in individuals receiving TD139, a galectin-3 inhibitor, compared to control subjects. The findings from this study suggest that changes in serum biomarkers, including platelet-derived growth factor-BB, fibrinogen activator inhibitor-1, CCL18, and YKL-40 in patients with idiopathic pulmonary fibrosis (IPF), are associated with the downregulation of Gal-3 (31).

In addition to TGF-β, CTGF, interleukin 13 (IL-13), and FGF are all involved in the fibrotic process associated with IPF. FG-3019 (pamrevlumab) is a human monoclonal antibody that inhibits CTGF. The phase II PRAISE trial (NCT01890265) indicated that FG-3019 slowed the decline in forced vital capacity (FVC) and improved diffusing capacity of the lungs for carbon monoxide (32). However, no evidence supporting the antifibrotic effectiveness of FG-3019 was obtained in the subsequent phase III trial. IL-13 activates fibroblasts, promotes extracellular matrix synthesis, and induces myofibroblast transformation and epithelial cell apoptosis through both TGF-β-dependent and non-dependent pathways (33, 34). Nevertheless, in a phase II trial, lebrikizumab, a monoclonal antibody targeting soluble IL-13, did not achieve the anticipated slowing of FVC decline (34). FGF signaling plays a significant role in the pathogenesis of IPF. This signaling pathway is activated through a group of cell surface receptors known as fibroblast growth factor receptors (FGFRs). Notably, FGF-2 expression is elevated in lung tissues affected by IPF, and it is essential for epithelial repair following bleomycin-induced injury in mice, although it does not directly participate in the fibrotic process (35). Similarly, FGF-1 levels are also increased in IPF lung tissue, where it inhibits transforming growth factor-beta 1 (TGF-β1)-stimulated myofibroblast differentiation and epithelial-mesenchymal transition (EMT), thereby functioning as an antifibrotic agent (36). Therefore, therapeutic strategies that target FGF signaling may prove to be effective in mitigating fibrosis.

Additionally, autotaxin inhibitors have emerged as a prominent trend in 2023. Lysophosphatidic acid plays a significant role in mediating epithelial cell apoptosis and fibroblast recruitment during pulmonary fibrosis in Hoyles et al. (36) and Funke et al. (37). Autotaxin, an enzyme responsible for the production of lysophosphatidic acid (38), is upregulated in patients with IPF, making it a potential target for novel therapeutic approaches. Ziritaxet, a new Autotaxin inhibitor, demonstrated encouraging results by reducing plasma lysophosphatidic acid concentrations in a Phase IIa study involving 23 patients with IPF (39). However, in the subsequent Phase III trial, Ziritaxet did not lead to improved clinical outcomes compared to placebo (40). IPF pathogenesis can be driven by lysophosphatidic acid (LPA), which signals through six LPA receptors (LPA1–6). Among these, LPA1 signaling plays a critical role in the development of fibrotic diseases. Admilparant (BMS-986278), an LPA receptor antagonist, has been evaluated in a large phase 2 trial. Phase 3 trials, ALOFT-IPF and ALOFT-PPF, comparing admilparant to placebo in patients with IPF and PPF, respectively, have been initiated (41). Additionally, bexotegrast (PLN-74809), a dual-selective inhibitor of avβ6 and avβ1 integrins, is expressed at low levels in normal lung tissue but upregulated in IPF patients. It may block TGF-β activation during fibrogenesis. In the INTEGRIS-IPF phase 2 trial (42), IPF patients were randomized to receive various doses of bexotegrast or placebo, showing favorable safety and tolerability up to 12 weeks. A phase 2b/3 program (BEACON-IPF) is underway to further assess its efficacy, tolerability, and safety in IPF patients (41).

In conclusion, IPF is a progressive and irreversible lung disease with an etiology that remains incompletely understood. Consequently, further research into the pathogenesis of IPF is imperative. As our understanding of the disease's pathogenesis advances, it will be possible to develop novel targeted therapies based on these research findings, which can then be progressively introduced into clinical trials. The initial exploration of IPF's pathophysiology reveals numerous potential therapeutic targets, including αvβ6 integrin, Pentraxin 2, Galectin-3, connective tissue growth factor (CTGF), interleukin-13 (IL-13), fibroblast growth factor (FGF), and autotaxin. Notably, we identify significant shortcomings in recent phase III clinical trials, wherein certain drugs demonstrated efficacy in preclinical and phase II trials but failed to achieve desired outcomes in phase III trials. Future research should prioritize the identification of novel therapeutic targets and expedite their progression to phase III clinical trials.

## Limitations and benefits

Our study provides a systematic overview of the global research advancements in targeted therapy for IPF over the past two decades. Nevertheless, it is important to acknowledge certain limitations. First, this review is limited to literature published in English, which may lead to the exclusion of important studies available in other languages. Second, data collection was conducted solely through the WOSCC database, potentially missing significant research accessible in other databases, such as PubMed and Embase. Third, bibliometric analyses typically depend on bibliographic indexes, which may not provide a comprehensive view of new publications when faced with imperfect or insufficient indexes. Fourthly, due to the continuous updating of database, recently published high-quality clinical studies may be underestimated for their unsatisfactory citations. Finally, we acknowledge that this study did not analyze the funding information associated with the publications included, and we plan to address this aspect in our future research to provide a more comprehensive understanding of the funding landscape related to this field.

## Conclusion

In conclusion, the exploration of targeted therapies for IPF is undergoing significant development, as evidenced by the growing annual number of pertinent publications. Notably, the United States leads in both the quantity and quality of research, exerting substantial influence on the trajectory of this field. Previous studies have delineated various therapeutic mechanisms and their associated targets. Currently, the focus has shifted toward phase III clinical trials, which are critical for the validation of these targeted therapies. It is expected that concepts, such as “optimization,” “resolution,” and “autotaxin inhibitors” will become increasingly prominent in future research endeavors.

## Data Availability

The original contributions presented in the study are included in the article/supplementary material, further inquiries can be directed to the corresponding author/s.
